# Resident Preferences for Telemedicine Services in China in the Digital Health Era: Mixed Methods Study

**DOI:** 10.2196/67390

**Published:** 2025-09-03

**Authors:** Maomin Jiang, Jian Zhao, Ranran Zhao, Jialiang Feng, Manli Gu, Meifang Yang, Zengming Ma

**Affiliations:** 1 School of Nursing Soochow University Suzhou China; 2 Department of Pediatrics The Affiliated Hospital of Southwest Medical University Luzhou China; 3 Sichuan Clinical Research Center for Birth Defects Luzhou China; 4 Department of Psychiatry The First Hospital of Hebei Medical University Shijiazhuang China; 5 School of Basic Medical Sciences Wuhan University Wuhan China; 6 School of Kangda Nanjing Medical University Lianyungang China; 7 School of Nursing Southwest Medical University Luzhou China; 8 Fujian Psychiatric Center Fujian Clinical Research Center for Mental Disorders Xianyue Hospital Affiliated to Xiamen Medical College Xiamen China

**Keywords:** telemedicine services, discrete choice experiment, resident preferences, consultation fee, privacy protection, scope of services

## Abstract

**Background:**

In the digital health era, telemedicine has become a key driver of health care reform and innovation globally. Understanding the factors influencing residents’ choices of telemedicine services is crucial for optimizing service design, enhancing user experience, and developing effective policy measures.

**Objective:**

This study aims to explore the key factors influencing Chinese residents’ choices of telemedicine services, including consultation fee, physician qualifications, appointment waiting time, scope of services, privacy protection, and service hours. The study also analyzes preference heterogeneity among residents with different demographic characteristics to provide scientific evidence for optimizing telemedicine services in the digital health era.

**Methods:**

This study used a mixed methods design combining qualitative interviews and a discrete choice experiment. Interviews identified key telemedicine attributes, informing the discrete choice experiment scenarios. Preferences and willingness to pay were analyzed using mixed logit and latent class models.

**Results:**

Residents’ preferences for telemedicine services were primarily shaped by the scope of services, appointment waiting time, and privacy protection, with substantial willingness to pay for more comprehensive, secure, and timely services. The optimal telemedicine services configuration—offering consultation plus prescription, high privacy, immediate access, 24-hour availability, and expert physicians—yielded a maximum willingness to pay of RMB 661.6 (a currency exchange rate of US $1=RMB 7.1803 is applicable). Latent class analysis revealed pronounced heterogeneity: while privacy and service scope remained universally prioritized, older, male, rural, and less-educated residents favored broader coverage, easier platforms, and lower costs; younger, female, and highly educated groups preferred faster, higher-quality, and more privacy-sensitive services.

**Conclusions:**

This study reveals key drivers and significant demographic heterogeneity in Chinese residents’ preferences for telemedicine services. Residents demonstrated a high willingness to pay for comprehensive services (eg, “consultation + prescription”), enhanced privacy protection, and shorter appointment waiting times. Additionally, the study innovatively identified 3 distinct resident profiles: “Diverse-Service-Oriented,” “Utility-Oriented,” and “Value-Oriented,” and proposed differentiated optimization strategies to effectively address diverse resident needs, thereby promoting equitable access and efficient adoption of telemedicine services.

## Introduction

In the digital health era, telemedicine has emerged as a pivotal force driving reform and innovation in global health systems [1], and with rapid advances in mobile internet, artificial intelligence (AI), and big data, telemedicine services is transitioning from traditional in-person visits to integrated telemedicine models [2]. Telemedicine significantly enhances service accessibility, reduces urban-rural and regional disparities, and offers patients personalized and precise health management options [3]; meanwhile, demographic aging, the high prevalence of chronic diseases, and growing public demand for health management have challenged traditional systems in terms of resource allocation, efficiency, and cost control, falling short of meeting the demand for high-quality care [4]. The COVID-19 pandemic further underscored telemedicine’s unique value in minimizing cross-infection risks and ensuring essential care, markedly accelerating its adoption [5,6], and governments and health care organizations worldwide have introduced supportive policies to fortify its strategic role in public health systems [7].

However, telemedicine services face numerous challenges despite their rapid growth. First, data security and privacy protection remain pressing issues, with the security of patients’ personal health information on telemedicine platforms receiving widespread attention. Unauthorized access and data breaches can have serious implications for patient privacy and health care decision-making [8]. The increasing risk of cyberattacks and hacking incidents could lead to large-scale leaks of sensitive data, undermining public trust in telemedicine. The implementation of technical measures such as data encryption, access control, and security protocols is still inadequate, necessitating stricter security measures and regulatory mechanisms [9]. Second, legal frameworks and industry standards are not well developed, with a lack of unified norms and regulatory mechanisms. Existing health care regulations primarily target traditional health care models, making it difficult to fully address the unique characteristics of telemedicine, which may lead to risks regarding health care quality and safety and even result in legal disputes. In particular, liability attribution and dispute resolution in telemedicine consultations and cross-regional health care services remain unclear, and these legal gaps may hinder the willingness of health care institutions and professionals to participate in telemedicine services, thus affecting the healthy development of the industry [10]. There are also disparities in the adoption of technology and user acceptance. Older adult populations and residents in remote areas may lack the necessary digital devices and web-based access or may find it challenging to use new technologies, exacerbating the digital divide and limiting the promotion and adoption of telemedicine among these groups [11]. Some patients exhibit low trust in telemedicine, primarily due to perceived risks regarding clinical effectiveness and concerns over privacy and data security, leading them to prefer traditional face-to-face consultations [12]. The integration of telemedicine services into existing health care systems faces multiple obstacles—namely, inadequate interoperability of health information systems, absence of unified electronic health records standards and data exchange protocols, and restricted data sharing among institutions—which undermines continuity and coordination of care, elevates the risk of redundant testing and medication errors, and increases patient burden and overall health care costs [13,14]. The engagement and professional competency of health care providers are also key factors affecting the quality of telemedicine services. Some health care providers lack sufficient awareness and training related to telemedicine, which may lead to variability in service quality. Additionally, telemedicine has increased the workload of health care professionals, making them more susceptible to burnout and safety risks [15]. Addressing these challenges is essential for telemedicine to achieve its full potential and contribute meaningfully to the transformation of health care delivery in the digital age.

In choosing telemedicine services, 4 interrelated factors are paramount: cost, platform security, scope and quality of care, and user experience. First, in the absence of standardized fees and insurance reimbursement, service price sensitivity varies across income groups, and high out-of-pocket costs deter low-income residents from adopting telemedicine [16]. Second, trust hinges on robust data security and privacy safeguards; any perceived vulnerability in protecting personal health data undermines user confidence [17]. Third, the breadth and professionalism of services depend on deep integration with existing health systems and the credentials of participating clinicians—limited scope or uneven provider qualifications weaken perceived effectiveness and reliability [18]. Finally, technical performance, ease of use, and response efficiency critically shape user experience, especially for older adults and rural populations, where complex interfaces or unstable systems can significantly reduce adoption willingness [19].

In contrast to existing studies that often examine telemedicine preferences through single-factor surveys or traditional questionnaires [20], this study is the first to use a discrete choice experiment (DCE) to Chinese residents’ preferences for telemedicine services, thereby accurately quantifying the relative importance and trade-offs among cost, platform security, scope and quality of services, and technological performance in realistic, multiattribute decision scenarios [21]. Unlike prior research primarily focused on isolated dimensions such as cost or accessibility, we comprehensively assess the interaction effects of multiple attributes and uncover significant heterogeneity in preference structures across income, age, and health status subgroups [22]. By centering on end user perspectives, our findings offer policy makers and service providers concrete, evidence-based recommendations for differentiated pricing strategies, enhanced data security measures, expanded telemedicine offerings, and improved user experience—thus facilitating the targeted adoption and sustainable growth of telemedicine in China [23]. Overall, this study makes a significant contribution to the large-scale design and optimization of digital health services by systematically segmenting user preferences through DCEs. By using advanced user segmentation techniques, our research not only quantifies the relative importance of key telemedicine service attributes but also identifies distinct preference profiles across diverse demographic groups. This approach enables the precise tailoring of digital health solutions to user needs, advancing the practice of precision health at scale. Importantly, the analytical framework and empirical findings presented in this study position the DCE as a practical, policy-enabling optimization tool for digital health. Policy makers and health system planners can use our results to allocate resources efficiently, design differentiated telemedicine packages, and formulate targeted interventions that enhance accessibility, equity, and user satisfaction. By integrating robust user segmentation into digital health policy, our work directly supports the evidence-based transformation and sustainable growth of digital health services in China and beyond.

## Methods

### Selection of Attributes and Levels

According to the definition of DCE and associated standards, it is crucial to ensure the scientific validity and rationality of the attributes and levels selected. This means that the chosen attributes should accurately reflect the key characteristics of telemedicine services, and participants must be able to fully understand the content of different combinations within the choice sets to make informed decisions. Additionally, the design should consider the suppressiveness of levels to ensure that respondents can effectively weigh their preferred options across different combinations [24]. Based on existing research, it is generally recommended to include 6-8 attributes in health care DCE studies [25], while the number of choice sets should be kept between 8 and 16 to ensure the validity and reliability of the experimental results [26].

To identify the most relevant attributes influencing residents’ preferences for telemedicine services, we adopted a mixed methods design that combined a PRISMA (Preferred Reporting Items for Systematic Reviews and Meta-Analyses)–guided scoping review, expert consultation, and the Model for Assessment of Telemedicine. We searched Web of Science, PubMed, and China National Knowledge Infrastructure for publications from January 2015 to June 2024, using keywords such as “telemedicine,” “online health services,” “resident preference,” and “discrete choice experiment.” After removing duplicates, 286 records underwent title and abstract screening, followed by full-text evaluation of 67 papers; ultimately, 43 studies met the inclusion criteria (Table S1 in Multimedia Appendix 1). Frequently identified attributes included consultation fee, physician expertise, scope of services, and data privacy. In parallel, we reviewed 12 local government reports and regulations issued by provincial and national health commissions (2020-2024), which emphasized price transparency, accessibility standards, and information security requirements. Using content analysis, 2 researchers (JF and MG) independently extracted and categorized candidate attributes, resolving discrepancies through discussion; Cohen κ coefficient (κ=0.81) was calculated to assess interrater reliability. Next, we conducted 2 rounds of semistructured interviews with 16 telemedicine stakeholders—including clinicians, platform managers, health economists, and patients—to validate, refine, and prioritize the attributes. Participants rated the relevance and feasibility of each candidate attribute on a 5-point Likert scale. The retention of attributes was determined based on both their frequency in the literature and the consensus among expert ratings (mean score of ≥4.0). Interview transcripts were analyzed using thematic analysis. Two independent researchers initially generated preliminary codes and iteratively refined the coding manual through multiple rounds of discussion, ultimately synthesizing similar codes into core themes. Data saturation was achieved as no new themes emerged after the second round of interviews. Finally, the Model for Assessment of Telemedicine framework was applied to map and ensure the conceptual coverage of multidimensional outcomes across 3 levels: preimplementation (eg, fee), multidisciplinary assessment (eg, physician qualification, waiting time, scope of services, and service hours), and transferability. These themes were systematically integrated with the results of the literature review and expert consultations to optimize and select DCE attributes, ensuring that the final attribute set was grounded in empirical evidence and comprehensively reflected stakeholder perspectives—thus going beyond simple descriptive triangulation. Ultimately, 7 attributes were finalized: fee, physician qualification, appointment waiting time, scope of services, platform usability, privacy protection, and service hours, with each attribute specified by 3-4 levels (Table 1). Explanations and expert assessments for DCE attributes are shown in Table S2 in Multimedia Appendix 1.

**Table 1:** Attributes and levels.

Attributes	Levels	Introduction to attributes
Physician qualifications	1. General practitioner	Physician’s professional background, experience, and qualifications
	2. Specialist	
	3. Chief physician	
	4. Renowned expert professor	
Appointment waiting time	1. Immediate consultation	Time from appointment to actual service received
	2. Waiting time <2 hours	
	3. Waiting time between 2 and 4 hours	
	4. Waiting time >4 hours	
Scope of services	1. Online consultation only	Type of medical services provided
	2. Consultation + prescription	
	3. Consultation + prescription + medication delivery	
	4. Consultation + prescription + medication delivery + follow-up	
Platform usability	1. Easy	User-friendliness of the telemedicine platform, including interface design and ease of use
	2. Moderate	
	3. Difficult	
Privacy protection	1. High	Measures for protecting user personal information and medical records on the platform
	2. Medium	
	3. Low	
Service hours	1. 24-hour service	Availability of medical service time slots
	2. Business hours service (9:00-17:00)	
	3. Limited hours service (evening or weekend)	
Fee (RMB^a^)	1. 50	Cost of using telemedicine services (RMB)
	2. 100	
	3. 150	
	4. 200	

^a^A currency exchange rate of US $1=RMB 7.1803 is applicable.

In each choice set, respondents were presented with 3 options, including 2 specific telemedicine service alternatives and an opt-out option (“none of the above”). This design allowed respondents to choose between the 2 service options or explicitly indicate that neither was appealing to them, thereby enabling a more comprehensive expression of their preferences. The inclusion of the opt-out option ensured a realistic assessment of respondents’ decision-making behaviors, allowing us to more accurately understand their preferences and choice tendencies. Table 2 provides an example of a choice set. This approach allowed for a more nuanced understanding of residents’ preferences, reflecting the diversity of decision-making processes in real-world scenarios. This process ensured the comprehensiveness and depth of our research, allowing the selected attributes and levels to accurately reflect the multidimensional factors considered by residents when choosing telemedicine services. By systematically integrating literature review and expert interviews, our study aims to offer theoretical support and practical guidance for understanding resident preferences in telemedicine services, thereby optimizing the design and promotion of telemedicine services.

**Table 2:** Example of a discrete choice set presented to respondents in the discrete choice experiment survey. Respondents were asked to select the most attractive option.

Attribute	Option A	Option B	Option C
Fee (RMB^a^)	200	150	None of the above
Physician qualifications	Renowned expert professor	General practitioner	None of the above
Appointment waiting time	Waiting time <2 hours	Waiting time >4 hours	None of the above
Scope of services	Consultation + prescription + medication delivery	Consultation + prescription + medication delivery	None of the above
Platform usability	Difficult	Moderate	None of the above
Privacy protection	Medium	Low	None of the above
Service hours	24-hour service	Limited hours service (evening or weekend)	None of the above

^a^A currency exchange rate of US $1=RMB 7.1803 is applicable.

### DCE Instrument Design

In our study, our team used a fractional factorial design based on the principles of orthogonality and level balance to determine the optimal number of telemedicine service options for residents. Given the considerable number of attributes and levels involved, a full factorial design would generate an overwhelming number of combinations. Specifically, our design included 7 attributes: 3 attributes with 3 levels each and 4 attributes with 4 levels each. Randomly selecting 1 level from each attribute results in 6912 possible combinations (calculated as 3^3^×4^4^). Such a large number of combinations would not only be impractical for data collection but could also lead to excessive cognitive burden on participants, potentially compromising data quality. To effectively manage these combinations, we opted for a commonly used fractional factorial design to construct the DCE choice sets. The fractional factorial method is based on 2 key principles: orthogonality and balance, which ensure independence among attribute levels and their even distribution throughout the experiment, thereby avoiding potential biases [27,28]. This approach allowed us to reduce participants’ cognitive load while maintaining the validity of the data. For practical implementation, we used SPSS 27 software (IBM Corp) to generate orthogonal designs that met the study’s requirements. This resulted in 32 valid options, with sets numbered 1-16 designated as set A and sets numbered 17-32 as set B. This arrangement enabled us to create 16 choice sets for participant evaluation. Furthermore, to reduce the burden on participants, these 16 choice sets were randomly divided into 2 separate questionnaires, each containing 8 choice sets. This design not only simplified the decision-making process for participants but also improved the completion rate and reliability of the data. To evaluate the statistical robustness of our design, we calculated the D-efficiency value, a metric used to assess how well the design minimizes the variance of parameter estimates. The D-efficiency of our study design was 89.46, indicating high statistical efficiency and reliability of the model estimation. Prior to the formal survey, we conducted a pilot test (n=35) and cognitive interviews to explore participants’ understanding of the attributes and the logic behind their choices. Based on feedback, we made appropriate adjustments to the attribute wording and questionnaire format to enhance readability and comprehension. We also assessed participants’ cognitive burden using a 5-point Likert scale. The average score was 2.34 (SD 0.71), suggesting that respondents generally found the task manageable. To assess the validity of the DCE questionnaire, we incorporated a clearly dominant choice set (commonly referred to as a “lure question”) into both questionnaires [29]. The “lure question” is similar to other choice sets in that it includes 2 alternatives. For attributes with nonquantifiable levels, we assigned the same levels to both option A and option B. For attributes with quantifiable levels, such as fees, option A was designed to be clearly superior to option B, and we chose the best possible levels for option A. If a respondent did not select option A, it would indicate that the respondent was not paying close attention or was not making choices carefully (Table 3). Only those participants who correctly selected the dominant option were included in the subsequent analysis, which effectively ensured data quality and eliminated random responses. Additionally, we calculated the minimum sample size required for the DCE study using the empirical rule proposed by de Bekker-Grob et al [30], following the formula:



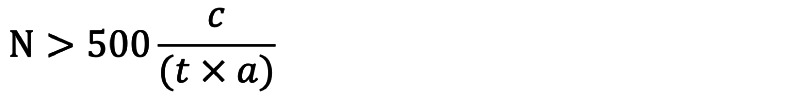



In the formula, *c* represents the largest number of levels for any attribute, meaning the attribute with the highest number of levels among all the considered attributes. The number of choice sets, *t*, refers to the total number of different choice tasks that each participant needs to evaluate, that is, the number of different choice scenarios that each participant is required to answer. *a* represents the number of alternatives per choice task, which indicates the number of options available for selection in each choice scenario (excluding the “none of the above” or “opt-out” option). This formula allows us to estimate the minimum required sample size to ensure statistical significance and generalizability of the study results. In this study, *c* was 4, *t* was 8, and *a* was 2, resulting in a calculated minimum sample size of 125. A total of 1186 valid samples were collected in this study, far exceeding the minimum required sample size. This ensured the accuracy and reliability of the experiment, providing a robust foundation for meaningful data analysis and interpretation.

**Table 3:** The choice set of lure question. Respondents were asked to select the most attractive option.

Attribute	Option A	Option B	Option C
Fee (RMB^a^)	50	200	None of the above
Physician qualifications	Renowned expert professor	General practitioner	None of the above
Appointment waiting time	Immediate consultation	Waiting time >4 hours	None of the above
Scope of services	Consultation + Prescription + Medication Delivery + Follow-up	Consultation + Prescription + Medication Delivery + Follow-up	None of the above
Platform usability	Easy	Difficult	None of the above
Privacy protection	Low	High	None of the above
Service hours	24-hour service	Limited hours service (evening or weekend)	None of the above

^a^A currency exchange rate of US $1=RMB 7.1803 is applicable.

### Data Collection

The study was conducted from September 1 to 20, 2024, using a multistage stratified sampling framework to ensure representativeness and generalizability. First, based on the National Bureau of Statistics’ delineation of eastern (highly developed), central (moderately developed), and western (less developed) regions, 3 provinces were purposively selected in each region: Fujian, Jiangsu, and Shandong in the east; Anhui, Heilongjiang, and Henan in the central area; and Shaanxi, Sichuan, and Guangxi Zhuang Autonomous Region in the west. Second, within each province, a key city—either the provincial capital or a major municipality—was selected: Xiamen (Fujian), Wuxi (Jiangsu), Yantai (Shandong), Xuancheng (Anhui), Harbin (Heilongjiang), Xinxiang (Henan), Xi’an (Shaanxi), Chengdu (Sichuan), and Liuzhou (Guangxi). Within each city, we divided the areas into 3 strata: central urban districts, suburban zones, and periurban or rural villages. From each stratum, 50 permanent residents were randomly selected using a synchronized random number generator applied to household rosters obtained from community health centers and family doctor registration systems. Trained enumerators conducted face-to-face surveys with randomized scheduling across different time slots to mitigate potential time-of-day and accessibility-related selection bias. During the survey implementation, we assigned 2-4 surveyors for each region, ensuring that each survey team was composed of experienced members. To enhance the effectiveness and consistency of the survey, all surveyors received standardized web-based training, focusing on the meaning of survey content, the proper way to complete questionnaires, and effective communication strategies with participants, aiming to minimize biases and errors caused by surveyors. To enhance the accuracy and completeness of our survey data, we implemented a “dual-interviewer and double-entry” protocol for each questionnaire: 2 uniformly trained interviewers conducted each interview in tandem or in alternation—one leading the interview and recording responses, the other independently reviewing and recording key items. Upon completion, both interviewers compared their records, with the survey supervisor adjudicating any discrepancies to ensure data consistency and validity. In this study, a total of 1298 questionnaires were distributed and 1186 valid questionnaires were collected, of which 112 excluded individuals were significantly lower in education (averaging middle school or below) and reported less prior exposure to telemedicine services. Additionally, their responses showed higher variance across choice tasks, reflecting random or inconsistent decision-making. We conducted a sensitivity analysis to test the robustness of our results. Comparisons of the core model estimates with and without the excluded data revealed no substantive differences in parameter significance or latent class segmentation. The D-efficiency changed by less than 2%, indicating model stability. Exclusion of inattentive respondents thus enhanced the internal consistency and interpretability of the findings.

This study established strict inclusion and exclusion criteria to ensure the validity of the data and the ethical conduct of the research. The inclusion criteria were as follows: (1) participants were at least 18 years of age; (2) participants had no cognitive impairments, ensuring that they could understand the content of the questionnaire; and (3) participants had a basic understanding of telemedicine services to ensure that they could make informed decisions regarding their preferences. The exclusion criteria included (1) questionnaires with numerous unanswered questions, indicating a lack of attentiveness in completing the survey; (2) incorrect responses to the lure question, suggesting that the participant’s responses might not be honest or consistent; (3) participants who were minors; (4) participants who could not understand the questions; (5) participants with severe mental illnesses; (6) participants who had previously taken part in similar studies; and (7) participants with cognitive impairments that prevented independent thinking.

### Ethical Considerations

To ensure ethical compliance, the first page of the survey questionnaire provided a detailed description of the study’s purpose and significance, emphasizing the participants’ autonomy in choosing whether to participate. Before completing the questionnaire, respondents were required to carefully read the informed consent form and confirm their understanding of its contents before being allowed to proceed. This process not only protected participants’ rights but also enhanced the transparency and credibility of the study. The questionnaire consisted of 3 sections. The first section collected demographic information, including gender, age, marital status, residence, education level, and monthly average income. This information facilitated the analysis of differences in telemedicine service preferences across various population groups. The second section focused on participants’ preferences for telemedicine services. In this section, we first presented a representative choice set along with a sample response to help participants understand the design and evaluation method of the DCE. Subsequently, participants were asked to evaluate 8 choice sets as part of the formal survey, followed by a choice set with a clearly dominant option, used as a lure question to assess the consistency and validity of participants’ responses. The study obtained informed consent from all participants and was conducted in accordance with the Declaration of Helsinki and was reviewed and approved by the medical ethics committee of Xiamen Xianyue Hospital (approval number 2024-KY-071), ensuring both ethical and scientific rigor in the research. Informed consent was obtained from all individual participants included in the study. Participants’ privacy and confidentiality were strictly protected throughout the research process.

### Data Analysis

Descriptive statistics were used to present the demographic characteristics of Chinese residents. The random utility theory provided the theoretical foundation for analyzing DCE data [31]. The DCE data were analyzed using a mixed logit (MXL) model in Stata 15 (StataCorp), where all attribute levels, except for cost, were dummy coded. This approach helped address the heterogeneity inherent in the MXL model and the independence of irrelevant alternatives assumption. Additionally, the nlcom command was used to calculate the willingness to pay (WTP) for given changes in levels across different scenarios, reflecting the preferences of Chinese residents for telemedicine services.

## Results

### Characteristics of Respondents

The sociodemographic characteristics of the study participants are shown in Table 4. A total of 1186 participants were included in the study, with a gender distribution of 56.41% (669) male and 43.59% (517) female. In terms of age, the majority of participants were aged between 18 and 30 years (436, 36.76%) and 31 and 44 years (361, 30.44%). Participants aged 45-60 years and older than 60 years accounted for 19.22% (228) and 13.58% (161), respectively. Regarding marital status, 71.33% (846) of participants had a partner, while 28.67% (340) were without a partner. In terms of place of residence, urban residents constituted 64.50% (765), while rural residents accounted for 35.50% (421). Regarding education level, the majority of participants had a medium education level (10-12 years), accounting for 43.59% (517). The remaining participants included those with lower education (≤9 years), comprising 17.12% (203), and those with higher education (>12 years), comprising 39.29% (466). Finally, in terms of monthly per capita disposable income, most participants had an income between RMB 2000 and 3500 (448, 37.77%; a currency exchange rate of US $1=RMB 7.1803 is applicable) and RMB 3501 and 5000 (376, 31.70%). Participants with incomes below RMB 2000 accounted for 10.46% (124), while those with incomes above RMB 6500 accounted for 7.25% (86).

**Table 4:** Characteristics of the study sample (N=1186).

Variable		Respondents, n (%)
**Sex**		
	Female	517 (43.59)
	Male	669 (56.41)
**Age (years)**		
	18-30	436 (36.76)
	31-44	361 (30.44)
	45-60	228 (19.22)
	>60	161 (13.58)
**Marital status**		
	Have a partner	846 (71.33)
	No partner	340 (28.67)
**Location**		
	Urban	765 (64.5)
	Rural	421 (35.5)
**Education level**		
	Low (≤ 9 years)	203 (17.12)
	Medium (10-12 years)	517 (43.59)
	High (>12 years)	466 (39.29)
**Monthly per capita disposable income (RMB** ^a^ **)**		
	<2000	124 (10.46)
	2000-3500	448 (37.77)
	3501-5000	376 (31.7)
	5001-6500	152 (12.82)
	>6500	86 (7.25)

^a^A currency exchange rate of US $1=RMB 7.1803 is applicable.

### MXL Model Results

Using the MXL model, we found a Δ*R*² of 0.412, with *P*<.001, indicating a good model fit. The results revealed that several factors significantly influenced residents’ decisions regarding telemedicine services. For consultation fee, an increase in cost was negatively associated with the likelihood of choosing telemedicine services, indicating that residents preferred lower-cost telemedicine options, with a β value of –.215 (*P*<.001). Regarding physician qualifications, using general practitioner as the reference group, residents’ preference for a specialist was not significant (β=–.010; *P*=.93), while they exhibited a stronger preference for a chief physician (β=.285; *P*=.01) and a renowned expert professor (β=.369; *P*<.001). In terms of appointment waiting time, using waiting time greater than 4 hours as the reference group, residents showed the highest preference for immediate consultation (β=.854; *P*<.001). Preference was also strong for waiting time less than 2 hours (β=.581; *P*<.001) and waiting time between 2 and 4 hours (β=.533; *P*<.001), suggesting that residents prefer shorter waiting times for telemedicine services. For scope of services, using web-based consultation only as the reference group, participants showed a high preference for the combination of consultation + prescription (β=.982; *P*<.001). The option of consultation + prescription + medication delivery also showed a preference (β=.177; *P*=.03), while consultation + prescription + medication delivery + follow-up did not show significance (β=.014; *P*=.88). Regarding privacy protection, using low privacy protection as the reference group, both high (β=.815; *P*<.001) and medium (β=.650; *P*<.001) levels of privacy protection had significant positive effects on preference, with a stronger preference for high privacy protection than for medium privacy protection. Finally, service hours had a significant impact on residents’ preferences. Compared with limited hours service, residents preferred 24-hour service (β=.539; *P*<.001) and business hours service (β=.494; *P*<.001), indicating that flexible service hours are an important factor in choosing telemedicine services. In conclusion, residents’ preferences for telemedicine services were influenced by multiple factors, including consultation fee, physician qualifications, appointment waiting time, scope of services, privacy protection, and service hours (Table 5).

**Table 5:** Residents’ preferences for telemedicine services. Model fit statistics: log-likelihood=–1120.45; Akaike information criterion=2316.90.

Variable			β		SE		*Z* value		*P* value		95% CI
Fee			–.215		0.051		–4.24		<.001		–0.315 to –0.115
**Physician qualifications**											
	General practitioner	1 [Reference]		N/A^a^		N/A		N/A		N/A	
	Specialist	–.010		0.107		–0.09		.93		0.220 to 0.201	
	Chief physician	.285		0.114		2.50		.01		0.062 to 0.508	
	Renowned expert professor	.369		0.103		3.58		<.001		0.167 to 0.570	
**Appointment waiting time**											
	Immediate consultation	.854		0.100		8.54		<.001		0.658 to 1.050	
	Waiting time <2 hours	.581		0.092		6.31		<.001		0.400 to 0.761	
	Waiting time between 2 and 4 hours	.533		0.084		6.33		<.001		0.368 to 0.698	
	Waiting time >4 hours	1 [Reference]		N/A		N/A		N/A		N/A	
**Scope of services**											
	Online consultation only	1 [Reference]		N/A		N/A		N/A		N/A	
	Consultation + prescription	.982		0.101		9.76		<.001		0.784 to 1.179	
	Consultation + prescription + medication delivery	.177		0.102		1.74		.03		0.023 to 0.377	
	Consultation + prescription + medication delivery + follow-up	.014		0.090		0.16		.87		0.163 to 0.191	
**Platform usability**											
	Easy	.051		0.092		0.55		.58		0.232 to 0.130	
	Moderate	.007		0.076		0.09		.93		0.143 to 0.156	
	Difficult	1 [Reference]		N/A		N/A		N/A		N/A	
**Privacy protection**											
	High	.815		0.092		8.89		<.001		0.635 to 0.994	
	Medium	.650		0.065		10.07		<.001		0.524 to 0.778	
	Low	1 [Reference]		N/A		N/A		N/A		N/A	
**Service hours**											
	24-hour service	.539		0.115		4.67		<.001		0.313 to 0.765	
	Business hours service	.494		0.091		5.44		<.001		0.316 to 0.671	
	Limited hours service	1 [Reference]		N/A		N/A		N/A		N/A	

^a^N/A: not applicable.

Table 6 shows the assessment of the relative importance of different attributes in respondents’ choices of telemedicine services. In a DCE, relative importance refers to the extent to which each attribute influences respondents’ preferences. This measure helps researchers understand the importance of each attribute in respondents’ decision-making processes. A larger weight indicates greater importance of that attribute in the decision process. The formula for calculating the weight is as follows: λᵢ = βᵢ /Σ*_j_* (β*_j_*), where λᵢ represents the relative weight of the i-th attribute, β*ᵢ* is the coefficient of the i-th attribute, calculated as the difference between the highest and lowest level coefficients for that attribute, and *j* represents the total number of attributes [32]. The attribute “Scope of Services” has the highest weight, indicating that it is the most important factor influencing respondents’ choices. “Appointment Waiting Time” and “Privacy Protection” also have relatively high weights, indicating that these attributes are highly valued by respondents. Conversely, “Platform Usability” has a relatively low weight, suggesting that it has a lesser impact on decision-making. These findings indicate that when selecting telemedicine services, respondents prioritize comprehensive services, short appointment waiting times, and privacy protection, while placing less emphasis on platform usability.

**Table 6:** Relative importance of different attributes in respondents’ choices of telemedicine services.

Category	Relative importance (%)
Service hours	14
Privacy protection	21
Platform usability	1
Scope of services	26
Appointment waiting time	22
Physician qualifications	10
Fee	6

### Willingness to Pay

Table 7 shows the WTP for changes in specific attribute levels, with WTP results indicating respondents’ preferences for telemedicine services in monetary terms. The findings reveal that, compared with other attributes, respondents were more willing to pay for improvements in Scope of Services, Privacy Protection, and Appointment Waiting Time. Specifically, respondents were willing to pay an additional RMB 197.727 for an upgrade from “Online Consultation Only” to “Consultation + Prescription” (*P*<.001; 95% CI 101.954-293.500), RMB 164.102 for high privacy protection instead of low privacy protection (*P*<.001; 95% CI 74.098-254.105), and RMB 116.852 for immediate consultation instead of waiting time greater than 4 hours (*P*<.001; 95% CI 51.236-182.667). In terms of Physician Qualifications, participants were willing to pay an additional RMB 57.396 to upgrade from a general practitioner to a chief physician (*P*=.02; 95% CI 8.878-105.914), and RMB 74.245 to upgrade to a renowned expert professor (*P*=.01; 95% CI 17.384-131.106). Regarding service hours, participants were willing to pay RMB 108.526 for a change from limited hours service to 24-hour service (*P*=.009; 95% CI 26.997-190.054) and RMB 99.412 for a change to business hours service (*P*<.001; 95% CI 39.855-158.968). The Platform Usability attribute, however, was found to be nonsignificant. According to the WTP results in Table 6, the optimal telemedicine service configuration—comprising “Consultation + Prescription” for Scope of Services, high Privacy Protection, Immediate Consultation for Appointment Waiting Time, 24-Hour Service for Service Hours, and Renowned Expert Professor for Physician Qualifications—had a maximum WTP of RMB 661.552.

**Table 7:** Willingness to pay estimates for attribute-level changes in telemedicine services.

Variable			Willingness to pay (RMB^a^)		SE		*Z* value		*P* value		95% CI
**Physician qualifications**											
	General practitioner	1 [Reference]		N/A^b^		N/A		N/A		N/A	
	Specialist	1.948		21.710		0.090		.93		–40.602 to 44.498	
	Chief physician	57.396		24.755		2.320		.02		8.878 to 105.914	
	Renowned expert professor	74.245		29.011		2.560		.01		17.384 to 131.106	
**Appointment waiting time**											
	Immediate consultation	116.952		33.529		3.490		<.001		51.236 to 182.667	
	Waiting time <2 hours	107.364		31.655		3.390		.001		45.322 to 169.407	
	Waiting time between 2 and 4 hours	74.245		29.011		2.560		.01		17.384 to 131.106	
	Waiting time >4 hours	1 [Reference]		N/A		N/A		N/A		N/A	
**Scope of services**											
	Online Consultation Only	1 [Reference]		N/A		N/A		N/A		N/A	
	Consultation + Prescription	197.727		48.865		4.050		<.001		101.954 to 293.500	
	Consultation + Prescription + Medication Delivery	2.828		18.423		0.150		.88		–38.936 to 33.281	
	Consultation + Prescription + Medication Delivery + Follow-up	35.721		21.412		1.670		.10		–6.247 to 77.689	
**Platform usability**											
	Easy	10.259		17.995		0.570		.57		–25.010 to 45.528	
	Moderate	1.327		15.347		0.090		.93		–31.406 to 28.752	
	Difficult	1 [Reference]		N/A		N/A		N/A		N/A	
**Privacy protection**											
	High	164.102		45.921		3.570		<.001		74.098 to 254.105	
	Medium	131.025		33.955		3.860		<.001		64.474 to 197.576	
	Low	1 [Reference]		N/A		N/A		N/A		N/A	
**Service hours**											
	24-hour service	108.526		41.597		2.610		.009		26.997 to 190.054	
	Business hours service	99.412		30.387		3.270		.001		39.855 to 158.968	
	Limited hours service	1 [Reference]		N/A		N/A		N/A		N/A	

^a^A currency exchange rate of US $1=RMB 7.1803 is applicable.

^b^N/A: not applicable.

### Latent Class Model

Table 8 shows the results of the latent class analysis using a 3-class model to segment residents’ preferences for telemedicine services. The optimal number of classes was determined by evaluating model fit statistics, including the Bayesian Information Criterion and entropy. The 3-class solution was selected because it achieved the lowest Bayesian Information Criterion (3450.23) and the highest entropy (0.72) among competing models, indicating a robust balance between model fit and classification accuracy, with all classes exhibiting significant cost sensitivity (β=–.015 to –.009; *P* ≤.026). Class 1 (388/1186, 32.7%), termed the “Diverse Service–Oriented” group, is defined by the strongest negative fee coefficient (β=–.015; *P*=.03) combined with very high positive coefficients for comprehensive service bundles such as consultation plus prescription plus medication delivery (β=2.857; *P*<.001) and follow‑up care (β=2.351; *P*<.001), moderate willingness to wait 2-4 hours (β=1.356; *P*=.001), preference for chief physicians (β=.668; *P*=.04), easy platform usability (β=1.042; *P*=.03), and high privacy protection (β=1.058; *P*=.03). Class 2 (662/1186, 55.8%), the Utility‑Oriented segment, shows moderate cost sensitivity (β=–.009; *P*<.001) alongside strong preferences for immediate consultation (β=1.187; *P*<.001), top-tier providers including chief physicians (β=.685; *P*<.001) and expert professors (β=.558; *P*=.001), consultation plus prescription (β=.642; *P*<.001), high privacy (β=1.274; *P*<.001), easy usability (β=.499; *P*=.001), and extended service hours such as 24‑hour access (β=.318; *P*=.007) or standard business hours (β=.310; *P*=.02). Class 3 (136/1186, 11.5%), the Value‑Oriented group, maintains similar fee sensitivity (β=–.010; *P*=.005) but places greatest emphasis on short waiting times under 2 hours (β=.517; *P*=.045), high‑value service combinations such as consultation plus prescription (β=.957; *P*=.001) and consultation plus prescription plus medication delivery (β=.864; *P*=.005), chief physicians (β=.567; *P*=.046), and elevated privacy protection (β=.826; *P*=.001). These segments illustrate that, although cost universally reduces telemedicine uptake, the scope of services, waiting time, provider qualification, usability, privacy, and service hours differentially drive choice across resident types.

**Figure 1:**
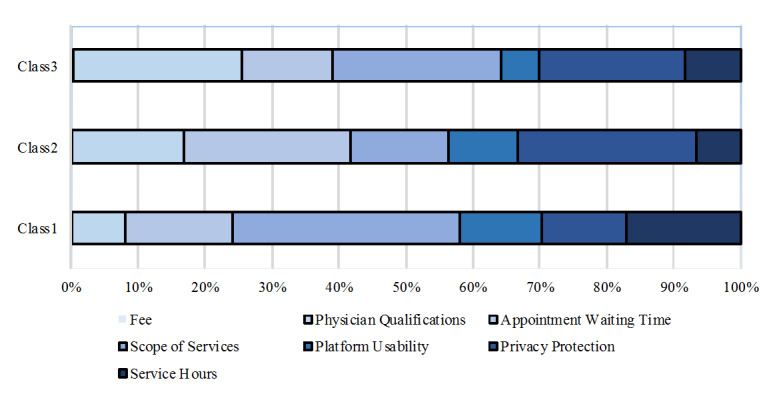
Assessment of relative importance of attributes for each class in latent class analysis. All 7 attributes were included in the model. The visual representation shows only 6 distinct bars per class because the coefficient for the fee attribute is relatively small compared with other attributes and thus may be visually obscured in the stacked format. However, fee was statistically significant and is discussed in the text.

The evaluation of the relative importance of different attributes for classes 1-3 reveals notable differences in attribute importance distribution among the 3 groups. For class 1, Privacy Protection, Scope of Services, and Cost accounted for the largest proportions, indicating that these respondents highly value privacy protection, prefer comprehensive services, and favor lower costs. In contrast, Platform Usability was the least important attribute for this group. Class 2 exhibited a more balanced distribution across all attributes, with slightly higher weights assigned to Scope of Services and Privacy Protection, suggesting a more balanced decision-making approach while still emphasizing comprehensive services and privacy. For class 3, Privacy Protection held the highest weight, followed by Scope of Services, indicating that privacy protection is the most critical consideration for these respondents, and they also have high expectations for comprehensive services. We defined the 3 classes based on the characteristics of the respondents’ preferences. Class 1, which exhibited a preference for a wide range of service content, was defined as Diverse Service–Oriented Class. Class 2, characterized by a strong preference for short waiting times, high-level physician qualifications, and high privacy protection, was defined as Utility-Oriented Class. Class 3, which preferred a combination of better service levels across multiple attributes, was defined as Value-Oriented Class. Overall, there are differences in preferences among the different classes when choosing telemedicine services. However, Privacy Protection and Scope of Services are consistently the most important factors across all classes, whereas Platform Usability is the least important for all groups. This finding suggests that, in designing telemedicine services, particular attention should be paid to enhancing privacy protection and the comprehensiveness of services to meet the needs of different respondent types, as illustrated in Figure 1.

Through multinomial logistic regression analysis, we examined the influence of demographic characteristics on residents’ latent class preferences for telemedicine services, using class 2 as the reference group. The results indicated that age, gender, and education were the primary factors affecting the likelihood of belonging to different preference groups. In the comparison between class 1 and class 2, age had a significant positive effect on belonging to class 1 (β =.137, *P*<.001; 95% CI 0.088-0.276). Education showed a significant negative effect on belonging to class 1 (β=–.453, *P*<.001; 95% CI –0.529 to –0.377), indicating that residents with lower educational attainment were more likely to belong to class 1. Place of residence also had a significant negative effect (β=–.176, *P*=.001; 95% CI –0.277 to –0.075), meaning that rural residents were more inclined to belong to class 1. Income did not have a significant effect on belonging to class 1 (*P*=.23). In the comparison between class 3 and class 2, age had a significant negative effect on belonging to class 3 (β=–.637, *P*<.001; 95% CI –0.722 to –0.553), indicating that younger residents were more likely to belong to class 3. For gender, females were more likely to belong to class 3 (β=–.350, *P*<.001; 95% CI –0.471 to –0.229). Education had a significant positive effect on belonging to class 3 (β=.107, *P*=.002; 95% CI 0.040-0.174), suggesting that residents with higher educational attainment were more likely to belong to class 3. Place of residence and income had no significant effects on belonging to class 3 (*P*>.05). Combining the preference characteristics of each class, it can be inferred that class 1 residents (older, male, with lower education, and from rural areas) tend to prioritize comprehensive Scope of Services and Platform Usability, are more cost-sensitive, and prefer services with longer waiting times. Class 2 (the reference group) showed moderate levels across demographic characteristics and exhibited preferences for highly qualified physicians, Immediate Consultation, and high Privacy Protection. Class 3 residents (younger, female, and with higher education) were more inclined to choose services with shorter waiting times, higher service quality, and richer Scope of Services, while placing a strong emphasis on Privacy Protection. These findings suggest significant differences in telemedicine service preferences among residents with different demographic characteristics. Older males with lower educational attainment are more likely to prefer basic and easy-to-use services, while younger, highly educated females tend to focus on service quality, efficiency, and privacy. These insights have important implications for developing targeted telemedicine service strategies, helping to enhance the specificity and effectiveness of telemedicine services for different population groups, as shown in Table 9.

**Table 8:** Latent class analysis of residents’ preferences for telemedicine services. Model fit statistics: entropy=0.72; Bayesian information criterion=3450.23.

Variable		Class 1 (388/1186, 32.7%)				Class 2 (662/1186, 55.8%)				Class 3 (136/1186, 11.5%)		
		β	*P* value	95% CI	β		*P* value	95% CI	β		*P* value	95% CI
Fee		–.015	.026	–0.029 to –0.002	–.009		<.001	–0.013 to 0.005	–.010		.005	–0.017 to –0.003
**Physician qualifications**												
	General practitioner	1.000	N/A^a^	N/A	1.000		N/A	N/A	1.000		N/A	N/A
	Specialist	.611	.27	–0.466 to 1.689	–.115		.52	–0.463 to 0.233	–.388		.17	–0.936 to 0.161
	Chief physician	.668	.04	0.037 to 1.372	.685		<.001	0.311 to 1.060	.567		.046	0.014 to 1.148
	Renowned expert professor	–.002	>.99	–0.873 to 0.869	.558		.001	0.226 to 0.890	.427		.13	–0.975 to 0.122
**Appointment waiting time**												
	Immediate consultation	.133	.58	–0.334 to 0.601	1.187		<.001	0.917 to 1.458	.252		.38	–0.313 to 0.817
	Waiting time <2 hours	.266	.29	–0.230 to 0.762	.782		<.001	0.479 to 1.085	.517		.045	0.012 to 1.047
	Waiting time between 2 and 4 hours	1.356	.001	0.575 to 2.138	.668		<.001	0.394 to 0.942	.217		.44	–0.331 to 0.765
	Waiting time >4 hours	1.000	N/A	N/A	1.000		N/A	N/A	1.000		N/A	N/A
**Scope of services**												
	Online consultation only	1.000	N/A	N/A	1.000		N/A	N/A	1.000		N/A	N/A
	Consultation + prescription	.005	.99	–0.629 to 0.639	.642		<.001	0.386 to 0.899	.957		.001	0.373 to 1.542
	Consultation + prescription + medication delivery	2.857	<.001	1.760 to 3.955	.317		.005	0.098 to 0.537	.864		.005	0.257 to 1.470
	Consultation + prescription + medication delivery + follow-up	2.351	<.001	1.742 to 2.961	–.058		.72	–0.377 to 0.260	.673		.02	0.091 to 1.255
**Platform usability**												
	Easy	1.042	.03	0.121 to 1.963	.499		.001	0.214 to 0.785	.218		.36	–0.248 to 0.683
	Moderate	.702	.01	0.144 to 1.260	.483		<.001	0.254 to 0.712	.088		.74	–0.428 to 0.604
	Difficult	1.000	N/A	N/A	1.000		N/A	N/A	1.000		N/A	N/A
**Privacy protection**												
	High	1.058	.03	0.134 to 1.982	1.274		<.001	0.957 to 1.591	.826		.001	0.332 to 1.320
	Medium	.196	.24	0.129 to 0.520	.747		<.001	0.553 to 0.941	.033		.92	–0.585 to 0.651
	Low	1.000	N/A	N/A	1.000		N/A	N/A	1.000		N/A	N/A
**Service hours**												
	24-hour service	.966	.004	0.304 to 1.629	.318		.007	0.086 to 0.550	.317		.19	–0.154 to 0.788
	Business hours service	–.481	.28	–1.361 to 0.399	.310		.02	0.060 to 0.680	.237		.41	–0.329 to 0.804
	Limited hours service	1.000	N/A	N/A	1.000		N/A	N/A	1.000		N/A	N/A

^a^N/A: not applicable.

**Table 9:** Results of the multinomial logistic regression analysis of the impact of demographic characteristics on residents’ latent class preferences for telemedicine services.

Variable		β	SE		*Z* value	*P* value	95% CI
**Comparison between class 1 and class 2 (class 2 as reference)**							
	Age (years)	.137		0.025	5.50	<.001	0.088 to 0.185
	Sex	.189		0.044	4.27	<.001	0.102 to 0.276
	Education level	–.453		0.039	–11.69	<.001	–0.529 to –0.377
	Location	–.176		0.051	–3.41	.001	–0.277 to –0.075
	Income	.058		0.048	1.21	.23	–0.036 to 0.153
	Constant	–2.951		0.533	–5.53	<.001	–3.996 to –1.906
**Comparison between class 3 and class 2 (class 2 as reference)**							
	Age	–.637		0.043	–14.72	<.001	–0.722 to –0.553
	Sex	–.350		0.062	–5.67	<.001	–0.471 to –0.229
	Education level	.107		0.034	3.14	.002	0.040 to 0.174
	Location	–.112		0.076	–1.47	.14	–0.262 to 0.037
	Income	–.058		0.070	–0.83	.41	–0.196 to 0.079
	Constant	12.199		0.897	13.59	<.001	10.440 to 13.958

## Discussion

### Principal Findings

The study results indicated that Chinese residents’ preferences for telemedicine services are influenced by multiple factors, including fee, physician qualifications, appointment waiting time, scope of services, privacy protection, and service hours. Compared with other attributes, participants were more willing to pay for enhancements in scope of services, privacy protection, and appointment waiting time. Specifically, they were willing to pay an additional RMB 197.727 to upgrade from “Online Consultation Only” to “Consultation + Prescription,” RMB 164.102 to attain high privacy protection from low privacy protection, and RMB 116.852 to reduce the appointment waiting time from greater than 4 hours to immediate consultation. The optimal telemedicine service configuration comprising “Consultation + Prescription” for scope of services, high privacy protection, immediate consultation for appointment waiting time, 24-hour service for service hours, and renowned expert professor for physician qualifications had a maximum WTP of RMB 661.552.

Latent class analysis of residents’ preferences for telemedicine services revealed significant heterogeneity across different resident groups. Factors such as cost, physician qualifications, appointment waiting time, scope of services, platform usability, privacy protection, and service hours had varying degrees of impact on different groups of residents. Further analysis using multinomial logistic regression suggested that older, male, less educated, and rural residents tend to prioritize comprehensive scope of services and platform usability, are more cost-sensitive, and prefer services with longer waiting times. In contrast, younger, female, and highly educated residents are more likely to prefer services with shorter waiting times, higher service quality, and enriched scope of services, while placing a high emphasis on privacy protection.

These findings indicate significant differences in telemedicine service preferences among residents with distinct demographic characteristics. Older males with lower education levels are more inclined to choose basic, easy-to-use services, whereas younger, highly educated females focus more on service quality, efficiency, and privacy. Understanding these preference differences has important implications for the development of targeted telemedicine service strategies, enabling tailored approaches that enhance service specificity and effectiveness for different population groups.

### From Uniformity to Precision: Segmenting Fee Sensitivity and Privacy Preferences for Tailored Telemedicine Optimization

Our DCE, augmented by latent class analysis, demonstrates that fee and privacy protection are the 2 most influential attributes shaping telemedicine uptake and that these drivers operate differently across distinct user segments, thereby demanding targeted policy strategies. First, all respondents exhibited aversion to high telemedicine fees echoing global evidence that insufficient reimbursement deters telemedicine adoption [33] and that most Chinese users are unwilling to pay beyond minimal out-of-pocket thresholds [34], but our segmentation clarifies who is most price-sensitive and for what they would be willing to pay more. The “Diverse‑Service” segment, comprising largely of older, lower-income, rural residents, was most deterred by cost and yet valued comprehensive service bundles, suggesting that low-cost basic packages perhaps underwritten by subsidized insurance schemes should be offered alongside premium add-ons to preserve service richness without pricing out vulnerable groups. In contrast, the “Utility-Oriented” segment characterized by working-age, midincome users demonstrated moderate fee sensitivity but expressed willingness to pay a premium for immediacy and provider expertise, implying that tiered pricing aligned with service speed (eg, fast-track access to senior clinicians) and qualifications could maximize uptake among this cohort. The “Value-Oriented” segment, skewing younger and more educated, balanced cost concerns with a strong preference for data security and rich service features; these users might accept higher fees for enhanced privacy guarantees and expanded telemedicine offerings, reflecting their readiness to invest in trustworthy, full-service telemedicine services, a pattern consistent with Italian findings that fee increases uniformly reduce choice but may be offset by perceived value [35].

Privacy protection emerged as the second critical driver: all classes prioritized robust data security, but the “Value-Oriented” segment exhibited the greatest sensitivity to privacy features, underscoring the need for advanced encryption, transparent data use policies, and regulatory oversight to build and maintain trust [36]. Frequent cybersecurity incidents have amplified patient fears around unauthorized health data access [37], and our theoretical application of the Health Belief Model suggests that perceived susceptibility to data breaches and perceived benefits of secure platforms jointly inform telemedicine adoption decisions. Policy must therefore mandate stringent privacy standards including regular third-party security audits and explicit user consent protocols and integrate these requirements into national telemedicine accreditation frameworks, thereby institutionalizing data protection as a core dimension of service quality.

Together, these insights translate into 4 concrete, evidence‑based recommendations for differentiated pricing, security, service provision, and user experience enhancements. Implement transparent, tiered pricing structures that offer subsidized basic telemedicine packages for cost-sensitive populations (eg, rural older adult), midrange options with expedited access for utility-oriented users, and premium bundles featuring extended services and guaranteed privacy protections for value-oriented adopters. Expand insurance coverage for telemedicine by incorporating eligible remote services into public and private reimbursement schemes, reducing out-of-pocket fees for low-income and high-need segments, and rewarding providers for serving underserved areas. Strengthen data security regulations by codifying advanced encryption, zero-trust architectures, and comprehensive privacy policies into law; require platforms to publish security certifications and privacy impact assessments, thereby enhancing transparency and accountability. Tailor user experience improvements via segmented interface design and education campaigns: simplify workflows and technical support for older, less digital-savvy users, offer fast-track scheduling and high-quality video for working-age professionals, and provide customizable privacy dashboards for younger, digitally literate users.

### Dual Engines of Scope and Speed: Segment-Based Pathways to Elevate Telemedicine Value

Our latent class analysis revealed that while all users derive value from a broad range of telemedicine offerings and timely access, the specific combinations of service scope and waiting-time preferences and their corresponding WTP vary markedly across the 3 latent segments we identified. The Diverse Service–Oriented segment, which is predominantly composed of older, lower‑income, and rural residents, displays an exceptionally high preference for an integrated care model encompassing not only web-based consultation but also prescription fulfillment, medication delivery, and structured follow-up. Although this group is the most cost-sensitive overall, it is willing to pay a moderate premium for a one-stop solution that minimizes logistic burdens, reflecting their limited access to traditional health care infrastructure. For this cohort, telemedicine platforms should bundle core services consultation, e‑prescribing, and remote follow-up into an affordable basic package, while offering add-on modules (eg, expedited medication dispatch or chronic care monitoring) at modest incremental fees. Aligning pricing tiers with these service bundles will allow operators to capture incremental WTP without alienating price‑vulnerable users [38].

In contrast, the Utility-Oriented segment characterized by working-age, midincome professionals places its highest value on rapid appointment access, exhibiting strong WTP for immediate or same-day teleconsultations. Our WTP estimates indicate that this group will tolerate elevated fees for accelerated response times far beyond what the Diverse Service–Oriented users find acceptable. To address this, telemedicine providers should implement service-level agreements guaranteeing predefined response windows, coupled with dynamic pricing that charges a premium for narrower waiting windows. Integrating intelligent scheduling algorithms, prioritization queues, and dedicated clinician “fast-track” pools staffed by senior physicians and supported by AI triage can ensure that this segment’s demand for prompt attention is met, while also maximizing revenue from users who equate time savings with improved health outcomes.

The Value-Oriented segment, composed largely of younger, highly educated urban residents, demonstrates a balanced but particularly strong inclination to pay for both comprehensive service scope and minimal waiting time, while also demanding the highest levels of data security. This cohort’s WTP peaks for service bundles that integrate full diagnostic workflows (consultation, prescription, delivery, and follow-up) within expedited access windows, signaling that they perceive telemedicine as a holistic health investment rather than a mere convenience. To capture this high-value market, platforms should offer premium “concierge” telemedicine plans, featuring guaranteed sub–15-minute waiting times, dedicated care navigators, extended follow-up analytics (eg, wearable data integration), and proactive outreach. These plans can be priced substantially above base service levels, reflecting the Value-Oriented segment’s willingness to invest in both speed and depth of service.

Across all segments, our findings underscore two critical imperatives for enhancing WTP through scope and timeliness optimization: (1) segmented service design that aligns bundles and response guarantees with distinct user preferences and WTP thresholds, and (2) flexible, transparent pricing frameworks that allow users to self-select appropriate service tiers without fear of hidden costs. Policy makers and insurers can facilitate these segmented offerings by endorsing reimbursement structures that reward providers for delivering bundled care services and for meeting defined response time benchmarks. For instance, value-based payment models could allocate higher reimbursements for telemedicine encounters that include follow-up coordination and medication adherence monitoring or that adhere to rapid response criteria.

Technologically, achieving these optimized scope-and-speed offerings requires robust platform architecture including AI-driven workload forecasting to align clinician availability with anticipated demand peaks, end-to-end encrypted communication channels to satisfy high privacy standards, and integrated logistics partnerships for same-day medication delivery. Equally important is ongoing user education, informing residents particularly in the Diverse Service–Oriented segment about the benefits of bundled telemedicine care and the trade-offs between service tiers. By communicating clearly how different packages map to clinical outcomes and convenience gains, platforms can enhance perceived value and nudge users toward higher-tier options when appropriate.

### From Credentials to Clicks: Segment-Driven Co-Optimization of Physician Expertise and Platform User Experience

Our latent class analysis has demonstrated clearly that preferences for physician qualifications and platform usability are not uniform but instead vary systematically across the 3 segments we identified, underscoring the need for differentiated strategies rather than one-size-fits-all solutions. The Diverse Service–Oriented segment—composed predominantly of older, less‑educated, and rural residents—exhibits lower baseline expectations for specialist credentials but places a high premium on ease of use and basic accessibility; for these users, platforms must prioritize streamlined interfaces, large‑font displays, and one-click workflows to reduce cognitive and operational barriers, while ensuring that a core cadre of qualified general practitioners is always available to build trust. In contrast, the Utility-Oriented segment, largely working-age, midincome individuals, values rapid, reliable access to seasoned physicians such as chief physicians and specialist consultants; this group will tolerate moderate fees in exchange for guaranteed appointment windows and clear indicators of provider credentials. Telemedicine services targeting this cohort should therefore institute tiered appointment pathways, with dedicated “priority lanes” for high‑qualification providers, backed by short wait‑time tiered service‑level agreements and transparent physician profiles. The Value‑Oriented segment skewing younger, female, and highly educated demands both top-tier credentials (renowned experts and professors) and a sophisticated, interactive user experience; they are willing to pay a premium for platforms that integrate advanced telemedicine features (eg, real-time video diagnostics and AI‑assisted triage) within a polished, intuitive user interface. For these high-value users, partnerships with leading academic medical centers can support expert-driven consultation services, while investment in user experience or user interface personalization (custom dashboards and preference-based content) will boost satisfaction and retention [39,40].

To translate these differentiated insights into policy and practice, we recommend 4 evidence-based actions. Segmentation-Driven Workforce Allocation: mandate that telemedicine providers maintain a minimum ratio of generalist-to-specialist clinicians aligned with user segment distribution ensuring that Diverse Service users always find accessible general practitioners, while Utility-Oriented and Value-Oriented users have clear options to consult higher-level providers. Tiered Service‑Level Agreements: require platforms to publish response time guarantees tied to fee tiers, such as basic plans with standard wait times and premium plans with expedited access to chief physicians, thereby empowering users to self-select according to WTP and need for professional expertise. Institutional Partnerships and Credential Transparency: encourage or subsidize collaborations between telemedicine platforms and top hospitals or medical schools to onboard renowned experts and professors and implement verified credential badges within the user interface to signal provider qualifications, directly addressing the Value‑Oriented segment’s demand for authority and trust. Usability Standards and Certification: develop national usability benchmarks for telemedicine interfaces covering navigation simplicity, accessibility features, and mobile responsiveness, and establish a certification program that signals compliance, reassuring the Diverse Service–Oriented and rural older adult users that platforms meet rigorously tested usability criteria.

### From One‑Size‑Fits‑All to Precision Insights: Demographic-Driven Stratified Telemedicine Strategies

Our DCE, augmented by latent class analysis, uncovers profound demographic heterogeneity in telemedicine preferences, exposing the need for finely tuned service strategies rather than broad, undifferentiated solutions. In the Diverse Service–Oriented cohort—comprising predominantly older, male, less-educated, and rural residents—the adoption of telemedicine depends mainly on basic ease of use and affordability; these users, often unfamiliar with digital interfaces and constrained by limited bandwidth or device access, exhibit a willingness to engage only if platforms deliver streamlined consultation and e‑prescription functions through an ultra-basic interface that minimizes clicks, text entry, and cognitive load, supplemented by robust offline support such as call center assistance and community health worker outreach to bridge digital literacy gaps [41-43]. Despite their pronounced cost sensitivity, they will devote moderate additional resources to receive integrated care bundles when offered in a highly transparent, insured, or subsidized pricing model that eliminates hidden fees and aligns with local reimbursement schemes. By contrast, the Utility-Oriented segment—largely working-age, midincome urban professionals—demonstrates stronger technology acceptance but demands uncompromising speed and clinical authority; this group exhibits an elevated willingness to pay a premium for guaranteed same day or less than ‑1‑hour access to senior physicians, coupled with clear on-‑platform display of provider credentials. They interpret telemedicine not merely as a convenience but as a credible substitute for in-person care when time is scarce. For them, a well-crafted service pathway that transparently ties fee tiers to response-time commitments, supported by intelligent scheduling algorithms and AI‑assisted triage to vet urgency, will command higher uptake and justify incremental fees.

Even more striking is the Value‑Oriented segment chiefly younger, female, and highly educated whose deep digital health literacy and elevated expectations lead them to prioritize comprehensive service scope, sophisticated platform usability, and rigorous data security measures in tandem. They will invest significantly more for packages that integrate advanced features such as real-time video diagnostics, wearable device data integration, proactive remote monitoring, and personalized health analytics, alongside end-to-end encryption and transparent privacy policies that assuage concerns over unauthorized use or breach of sensitive health information [37]. For this segment, telemedicine is perceived as a holistic health investment; they seek not only functional excellence but also an engaging user experience characterized by customizable dashboards, interactive health education modules, and seamless interoperability with other digital health tools.

These 3 latent classes together illustrate that demographic factors, such as age, gender, education, and urbanicity, intersect with technology acceptance, health needs, and socioeconomic capacity to produce starkly divergent telemedicine WTP profiles. The Diverse Service group will trade simplicity for affordability, the Utility-Oriented will exchange higher fees for speed and expertise, and the Value-Oriented will pay top dollar for both breadth and depth of digital health services underpinned by robust privacy safeguards. This granular understanding enables policy makers and service providers to craft nimbly differentiated telemedicine offerings that match each segment’s WTP and preference structure: ultra-basic, subsidized bundles delivered via simplified user interfaces for the digitally underserved; tiered, rapid-response plans with transparent clinician credentials for time-pressed professionals; and premium, concierge-style packages combining AI-driven innovation and stringent data security for digitally savvy enthusiasts. Embedding these differentiated strategies within adaptive pricing frameworks, value-based reimbursement models, and inclusive digital literacy initiatives will transform telemedicine from a one-size-fits-all novelty into an equitable, sustainable pillar of China’s health care ecosystem ensuring that every resident, regardless of demographic background, can confidently access, afford, and benefit from digital health services.

### Limitations

While this study provides valuable insights into residents’ preferences for telemedicine services, several limitations must be acknowledged. First, the representativeness of the sample may be limited, as participants were primarily recruited from specific regions and may not fully reflect the geographic, cultural, and socioeconomic diversity of the entire country. This limitation may affect the external validity and generalizability of the findings. Second, the DCE methodology has inherent limitations. Although DCEs are designed to mimic real-world decision-making, responses are elicited in hypothetical scenarios, introducing potential hypothetical bias and social desirability bias. Moreover, the analysis is based on the assumption of utility maximization, which may not always align with actual respondent behavior in real-life contexts. The WTP estimates derived from DCEs are based on stated rather than revealed preferences and may diverge from actual economic decisions made by individuals. In addition, the study focused primarily on selected attributes such as cost, platform security, scope of services, and quality, and may have omitted other relevant factors that influence preferences. Unmeasured confounding variables may also affect the observed associations, despite attempts to control for certain demographic factors in regression analyses. Finally, the cross-sectional design of this study precludes the assessment of changes in preferences over time. As technology and policy continue to evolve, resident preferences for telemedicine services may shift, and the findings of this study may not capture future trends. Future research should consider longitudinal designs and incorporate emerging service models to better understand evolving user preferences.

### Conclusions

Using an innovative mixed methods design combining a DCE and latent class analysis, this study uniquely identifies key drivers and demographic heterogeneity influencing Chinese residents’ preferences for telemedicine services. Residents demonstrated a high WTP for comprehensive services (eg, “Consultation + Prescription”), enhanced privacy protection, and shorter waiting times. Three distinct preference profiles were innovatively delineated: “Diverse Service–Oriented,” “Utility-Oriented,” and “Value-Oriented.” Tailored service strategies are recommended accordingly—offering affordable, user-friendly service packages for older, less-educated residents; rapid access to specialist services for working-age professionals; and premium, secure, and comprehensive telemedicine solutions for young, highly educated users. These findings provide novel, actionable evidence to inform differentiated and targeted telemedicine service optimization, promoting equitable access and efficient uptake of telemedicine services in China.

## Appendix

Multimedia Appendix 1 Findings and expert judgment.

## Abbreviations

DCE: discrete choice experiment
MXL: mixed logit
PRISMA: Preferred Reporting Items for Systematic Reviews and Meta-Analyses
WTP: willingness to pay

## Data Availability

The datasets generated and analyzed during this study are not publicly available due to privacy and confidentiality agreements with the participants. However, data may be available from the corresponding author on reasonable request and upon approval by the relevant ethics committee.
