# Supplementary material for: Resident Preferences for Telemedicine Services in China in the Digital Health Era: Mixed Methods Study
Source: J Med Internet Res. 2025 Sep 3;27:e67390. doi: 10.2196/67390 (PMC12452206; doi:10.2196/67390)
Supplement: Multimedia Appendix 1 [file jmir_v27i1e67390_app1.docx]

**Table S1.** Summary of literature findings and expert judgment.

| Topic | Key findings | Expert judgment |
| --- | --- | --- |
| Data Security & Privacy[8-9] | Patient health data on telemedicine platforms face cyberattack and breach risks; existing encryption and access controls are inadequate. | Embed end-to-end encryption, multi-factor authentication, and real-time security monitoring in platform design; commission periodic third-party security audits and disclosure. |
| Legal & Regulatory Framework [10] | Cross-regional liability in telemedicine is unclear; lack of unified standards and dispute mechanisms raises medico-legal risks . | Enact national telemedicine laws clarifying cross-province practice boundaries and liability; establish dedicated telemedicine arbitration bodies. |
| Digital Divide & Accessibility [11] | Elderly and rural residents lack devices and digital skills, limiting telemedicine uptake. | Strengthen rural/community internet infrastructure; launch 'Digital Health for Seniors' and 'Rural Telemedicine Experience' programs. |
| System Interoperability [12-14] | Health information systems lack unified EHR standards and data-exchange protocols; limited data sharing causes redundant tests and medication errors. | Promote national EHR interoperability standards, pilot FHIR in provincial centers, and extend to primary care facilities in phases. |
| Physician Qualifications & Depth [15,18] | Physician qualification level significantly impacts patient choice; higher credentials yield greater trust. | Clearly display physician credentials on platforms; implement differential pricing and subsidies to attract experts. |
| User Experience & Technical Performance [19] | Complex interfaces, unstable systems, and slow responses deter elderly and rural users . | Adopt user-centered design (large fonts, one-click call, voice prompts) and optimize backend for high availability and low latency. |
| Cost & Insurance Coverage [16] | Lack of standardized fees and insurance reimbursement; high out-of-pocket costs deter low-income groups. | Develop tiered pricing with insurance reimbursement; provide targeted subsidies for low-income and chronic disease patients. |

**Table S2.** Findings and expert judgment of DCE attributes.

| Attribute | Level | Findings | Expert judgment |
| --- | --- | --- | --- |
| Physician Qualifications | 1. General Practitioner  2. Specialist  3. Attending Physician  4. Renowned Expert Professor | Higher qualification levels significantly increase the probability of choice; expert professor level commands the highest premium. | Platforms should prioritize inclusion and display of high-qualification physicians, and implement subsidies and tiered pricing for expert services to guide resource allocation. |
| Waiting Time | 1. Immediate  2. <2 hours  3. 2-4 hours  4. >4 hours | Each increase in waiting time level decreases choice probability by ~15%; <2-hour slots are most preferred. | Recommend implementing time-slot reservations and virtual waiting rooms to reduce perceived wait times. |
| Scope of Services | 1. Consultation only  2. +Prescription  3. +Prescription + Medication Delivery  4. +Prescription + Delivery + Follow-up | Value-added services (delivery, follow-up) significantly boost preference; the full package enjoys a 25% preference premium over consultation only. | Encourage platforms to develop integrated service loops by linking logistics and community health resources for last-mile management. |
| Platform Usability | 1. Easy  2. Moderate  3. Difficult | Each drop in usability level reduces choice probability by over 10%; seniors are particularly sensitive to ease of use. | Adopt user-centered design: large fonts, one-click call, voice/icon guidance to enhance accessibility. |
| Privacy Protection | 1. High  2. Medium  3. Low | A one-level drop in privacy protection reduces preference score by ~20%. | Platforms should disclose security certifications, regularly commission third-party security assessments, and publicize results. |
| Service Hours | 1. 24-hour  2. Business hours (9:00-17:00)  3. Limited hours (Evening/Weekend) | 24-hour service significantly outperforms business hours, especially for urgent needs at night. | Recommend extending night and weekend services where resources allow and optimizing staffing with intelligent triage. |
| Fee (RMB) | 1. ¥50  2. ¥100  3. ¥150  4. ¥200 | Each ¥50 increase in fee reduces choice probability by ~12%; sensitivity is highest below ¥100. | Implement tiered pricing within cost constraints and integrate with insurance/subsidies to lower barriers for low-income groups. |
